# Stimulation of the calcium‐sensing receptor induces relaxations of rat mesenteric arteries by endothelium‐dependent and ‐independent pathways via BK_Ca_ and K_ATP_ channels

**DOI:** 10.14814/phy2.15926

**Published:** 2024-01-28

**Authors:** Simonette R. E. Carlton‐Carew, Harry Z. E. Greenberg, Eleanor J. Connor, Pooneh Zadeh, Iain A. Greenwood, Anthony P. Albert

**Affiliations:** ^1^ Vascular Biology Research Section, Molecular & Clinical Sciences Research Institute St. George's University of London London UK

**Keywords:** BK_Ca_ channels, calcium‐sensing receptor, endothelial cells, K_ATP_ channels, nitric oxide, perivascular nerves, vascular smooth muscle

## Abstract

Stimulation of the calcium‐sensing receptor (CaSR) induces both vasoconstrictions and vasorelaxations but underlying cellular processes remain unclear. This study investigates expression and effect of stimulating the CaSR by increasing external Ca^2+^ concentration ([Ca^2+^]_o_) on contractility of rat mesenteric arteries. Immunofluorescence studies showed expression of the CaSR in perivascular nerves, vascular smooth muscle cells (VSMCs), and vascular endothelium cells. Using wire myography, increasing [Ca^2+^]_o_ from 1 to 10 mM induced vasorelaxations which were inhibited by the calcilytic Calhex‐231 and partially dependent on a functional endothelium. [Ca^2+^]_o_‐induced vasorelaxations were reduced by endothelial NO synthase (eNOS, L‐NAME) and large conductance Ca^2+^‐activated K^+^ channels (BK_Ca_, iberiotoxin), with their inhibitory action requiring a functional endothelium. [Ca^2+^]_o_‐induced vasorelaxations were also markedly inhibited by an ATP‐dependent K^+^ channel (K_ATP_) blocker (PNU37883), which did not require a functional endothelium to produce its inhibitory action. Inhibitor studies also suggested contributory roles for inward rectifying K^+^ channels (K_ir_), Kv7 channels, and small conductance Ca^2+^‐activated K^+^ channels (SK_Ca_) on [Ca^2+^]_o_‐induced vasorelaxations. These findings indicate that stimulation of the CaSR mediates vasorelaxations involving multiple pathways, including an endothelium‐dependent pathway involving NO production and activation of BK_Ca_ channels and an endothelium‐independent pathway involving stimulation of K_ATP_ channels.

## 1 INTRODUCTION

The calcium‐sensing receptor (CaSR), a member of the Class C family of G‐protein‐coupled receptors, is activated by an increase in extracellular Ca^2+^ concentration ([Ca^2+^]_o_) to maintain plasma Ca^2+^ homeostasis by regulating parathyroid hormone secretion from the parathyroid gland which controls intestinal Ca^2+^ absorption, renal Ca^2+^ excretion, and bone remodeling (Brown & MacLeod, 2001; Hannan et al., 2018). There is increasing evidence that the CaSR is also present in tissues not normally associated with regulating plasma Ca^2+^ homeostasis such as blood vessels (Guo et al., 2018; Smajilovic et al., 2011; Weston et al., 2011). Moreover, recent findings indicate that tissues previously considered non‐calciotropic like vascular smooth muscle may modulate Ca^2+^ homeostasis (Hannan et al., 2018; Schepelmann et al., 2022). In the presence of closely regulated plasma Ca^2+^ levels, stimulation of the CaSR is considered possible in the vasculature as localized [Ca^2+^]_o_ is likely to vary sufficiently due to active cellular Ca^2+^ extrusion mechanisms such as the plasmalemmal Ca^2+^‐ATPase and Na^+^‐Ca^2+^ exchanger on vascular smooth muscle cells (VSMCs) and vascular endothelial cells (VECs) (Dora et al., 2008).

In the vasculature, the CaSR is proposed to be functionally expressed in perivascular nerves, VSMCs, and VECs to regulate vasorelaxations (Awumey et al., 2008, 2013; Bukoski et al., 1997; Greenberg et al., 2017, 2019; Greenberg, Jahan, et al., 2016; Greenberg, Shi, et al., 2016; Ishioka & Bukoski, 1999; Loot et al., 2013; Mupanomunda et al., 1998, 1999; Tang et al., 2016; Thakore & Ho, 2011; Wang & Bukoski, 1998; Weston et al., 2005, 2008) vasoconstrictions (Li et al., 2011; Schepelmann et al., 2016; Wonneberger et al., 2000), and biphasic responses (Ohanian et al., 2005) involving diverse cellular mechanisms. As such, the CaSR may be a therapeutic target for cardiovascular conditions involving dysfunction of vascular tone such as hypertension and septic shock (Guo et al., 2018; Sood et al., 2022).

Stimulation of the CaSR on perivascular nerves is reported to induce relaxation of rat mesenteric arteries via endothelium‐dependent and ‐independent pathways involving synthesis and release of vasoactive lipids, such as anandamide and cytochrome P_450_ metabolites, which activate large conductance Ca^2+^‐activated K^+^ channels (BK_Ca_) in adjacent VSMCs to produce membrane hyperpolarization and subsequent relaxation (Awumey et al., 2008; Bukoski et al., 1997, 2002; Ishioka & Bukoski, 1999; Mupanomunda et al., 1998, 1999; Wang & Bukoski, 1998). In addition, stimulation of the CaSR in VECs leading to activation of intermediate Ca^2+^‐activated K^+^ channels (IK_Ca_) and hyperpolarization and relaxation have also been shown in rat mesenteric arteries (Thakore & Ho, 2011; Weston et al., 2005, 2008). Our studies in mouse and rabbit mesenteric arteries suggest that activation of the CaSR on VECs induces relaxations via two separate endothelium‐dependent pathways involving stimulation of heteromeric TRPV4‐TRPC1 channels and nitric oxide (NO) generation which activates BK_Ca_ channels in VSMCs and stimulation of IK_Ca_ channels which produce endothelium‐derived hyperpolarization of VSMCs (Greenberg et al., 2017, 2019; Greenberg, Jahan, et al., 2016; Greenberg, Shi, et al., 2016). CaSR‐induced activation of Gq‐mediated pathways in VSMCs is also linked to vasoconstriction (Li et al., 2011; Wonneberger et al., 2000). Interestingly, prevention of CaSR‐mediated vasorelaxations by removal of a functional endothelium or a combined inhibition of endothelium NO synthesis and IK_Ca_ channel activity revealed CaSR‐mediated vasoconstrictions indicating that CaSR‐induced responses in a vascular bed are likely to be balance between relaxant and contractile mechanisms (Greenberg, Jahan, et al., 2016; Greenberg, Shi, et al., 2016).

Based upon different findings of the studies highlighted above in rat, rabbit, and mouse mesenteric arteries, the present study studied in further detail the role of the endothelium, NO production, and different K^+^ channel subtypes in CaSR‐mediated relaxations in rat mesenteric arteries. Our results show expression of the CaSR in perivascular nerves, VSMCs, and VECs, and that CaSR‐induced vasorelaxations involve multiple pathways involving an endothelium‐dependent pathway involving NO synthesis and activation of BK_Ca_ channels in VSMCs and an endothelium‐independent pathway involving ATP‐dependent K^+^ channels (K_ATP_) in VSMCs. This is the first time that K_ATP_ channels have been linked to mediating CaSR‐mediated vasorelaxations.

## 2 MATERIALS AND METHODS

### 2.1 Ethical approval

All animal procedures were carried out in accordance with guidelines laid down by St. George's, University of London Animal Welfare Committee, and conform with the principles and regulations described by the Service Project License: 70/8512 and to the principles and regulations described by Grundy (2015). Male Wistar rats (aged 10–12 weeks and weighing between 210 and 280 g) were used for the purpose of the study. Rats were supplied from Charles River Ltd (Margate, Kent, UK) and housed and maintained in standard‐sized plastic cages at the Biological Research Facility at St. George's, University of London under a 12:12 h light/dark photocycle at 18–22°C and 50 ± 10% relative humidity, with water and laboratory rodent diet and freshwater (Specialist Dietary Services, UK) available ad libitum.

Rats were culled by cervical dislocation and death was confirmed by severance of the femoral artery in accordance with the Schedule I of the UK Animals (Scientific Procedures) Act of 1986. First‐order branches of superior mesenteric artery were dissected and cleaned of adherent tissue in ice‐cold normal Krebs–Henseleit physiological salt solution (PSS) containing (mM): NaCl 118, KCl 4.7, MgSO_4_ 1.2, KH_2_PO_4_ 1.2, NaHCO_3_ 25, CaCl_2_ 1, D‐glucose 10.

### 2.2 Cell preparation

Isolated cells were prepared using Hanks' Balanced Salt Solution (HBSS) (Thermo Scientific, UK). To isolate VECs, dissected first‐order arteries were placed in 50 μM CaCl_2_ HBSS at 37°C for 5 min, placed into a 1 mg/mL collagenase solution at 37°C for 15 min, and then subsequently washed in fresh solution at 37°C for 10 min. Vessels were triturated in fresh 50 μM CaCl_2_ HBSS and the cell‐containing solution was collected and centrifuged at 1000 rpm for 1 min. The supernatant was removed, and cells were re‐suspended in 0.75 mM CaCl_2_ HBSS, plated onto coverslips, and left at 4°C for 1 h before use. To isolate VSMCs, a similar technique was used except initially the endothelium was gently removed from first‐order arterial segments with a cotton bud, and vessels were placed into 50 μM CaCl_2_ HBSS containing 0.5 mg/mL collagenase and 0.25 mg/mL proteases at 37°C for 1 h.

### 2.3 Immunofluorescence

#### 2.3.1 Isolated cells

Isolated VECs and VSMCs were fixed with 4% (v/v) paraformaldehyde (PFA) in phosphate‐buffered saline (PBS) for 10 min, washed three times with PBS, permeabilized with PBS containing 0.1% (v/v) Triton for 10 min, and then washed three times with PBS, all at room temperature. Fixed cells were then incubated in a humidity chamber in 1% (v/v) donkey serum in PBS with 0.1% (v/v) Tween 20 for 2 h. VECs were incubated overnight at 4°C with rabbit anti‐CD31 antibody (1:100) and mouse anti‐CaSR antibody (1:100), and VSMCs were incubated overnight at 4°C with rabbit anti‐α‐actin antibody (1:000) and mouse anti‐CaSR antibody (1:100) in PBS containing 1% donkey serum. The cells were then washed three times with PBS and incubated with secondary antibodies conjugated to a fluorescent probe for 1 h (Alexa Fluor 555/568‐conjugated donkey anti‐mouse antibody [1:500] and Alexa Fluor 488‐conjugated donkey anti‐goat antibody [1:500] and Alexa Fluor 546‐conjugated donkey anti‐rabbit antibody [ThermoFisher Scientific, Walham, MA, USA]). Unbound secondary antibodies were removed by washing with PBS, and then slides were bathed in Duolink® In situ mount with 4′,6‐diamidino‐2‐phenylindole (DAPI) for fluorescence protection and nuclei staining (Sigma–Aldrich, Sigma Chemical Co., Poole, UK). Cells were imaged using a Zeiss LSM 510 laser scanning confocal microscope (Carl Zeiss, Jena, Germany). Control experiments in which primary or secondary antibodies were omitted were carried out alongside test conditions. Excitation was produced by 488 nm, 546 nm, or 555 nm lasers and delivered to the specimen via a Zeiss Apochromat x63 oil‐immersion objective. Two‐dimensional immunofluorescent images cut horizontally through the middle of cells were captured, and raw confocal imaging data were processed using Zeiss LSM 510 software (release 3.2; Carl Zeiss). Final images were produced using PowerPoint (Microsoft Office, Microsoft, Richmond, WA, USA).

#### 2.3.2 Arterial segments

Segments from first‐order vessels were fixed with 4% (w/v) PFA in PBS at room temperature for 1 h and then washed in PBS three times for 10 min each. Arteries were then incubated in PBS containing 30% (w/v) sucrose overnight at 4°C for cryoprotection. The arteries were transferred to a cryomold mold with the vessel lumen positioned perpendicular to the sectioning surface of the mold and embedded in optimal cutting temperature (OCT) polymer compound, placed in dry ice containing ethanol to freeze the OCT. Molds were stored at −75°C until use. Cryomold molds were secured to a frozen sectioning chuck, with the sectioning surface orientated upwards parallel to surface of the chuck, at −55°C. Sectioning was conducted once the mold reached −30°C to obtain 10 μm thick cross‐sections of artery in a microtome‐cryostat (Thermo Scientific HM525 MX, UK). Sections were mounted onto SuperFrost adhesive microscope slides. After sectioning, the slides were air‐dried for 60 min and rehydrated with PBS at room temperature for 10 min each.

Arterial sections were incubated in 2% (v/v) donkey serum in PBS with 0.2% (v/v) Triton for 2 h. Mouse anti‐CaSR antibody (1:100), rabbit anti‐α‐actin antibody (1:100), rabbit anti‐CD31 antibody, rabbit anti‐synaptophysin (1:100), and the rabbit anti‐GPRC6A (1:100) were dissolved in PBS containing 2% (v/v) donkey serum and 0.2% (v/v) Triton and pipetted (100 μL each) onto the microscope slides containing artery sections and placed at 4°C overnight. The next day, the microscope slides were washed three times for 10 min each with PBS and the secondary antibodies were added (Alexa Fluor 555/568‐conjugated donkey anti‐mouse antibody [1:500], Alexa Fluor 488‐conjugated donkey anti‐goat antibody [1:500], Alexa Fluor 488‐conjugated donkey anti‐rabbit antibody, and Alexa Fluor 546‐conjugated donkey anti‐rabbit antibody) dissolved in PBS containing 2% (v/v) donkey serum and 0.2% (v/v) Triton, and placed in a humidity chamber with an opaque cover and left in a dark room for 2 h at room temperature. Control experiments in which primary or secondary antibodies were omitted from the protocol were carried out alongside test conditions. Microscope slides were washed in PBS three times at 10 min each and bathed in Duolink® In situ mount with DAPI for fluorescence protection and nuclei staining (Sigma–Aldrich, Sigma Chemical Co., Poole, UK). Coverslips were sealed with nail polish in preparation to be imaged. Fluorescent and brightfield images of arterial sections were captured using the Zeiss LSM510 META Inverted confocal scanning laser microscope. Once the images were captured, co‐localization of targeted proteins was represented by overlapping the individual immunofluorescence images.

### 2.4 Isometric tension recordings

Effects of stimulating the CaSR on vascular contractility were investigated using wire myography. Vessel segments from first‐order arteries of 2 mm in length were mounted in a wire myograph (Model 610 M; Danish Myo Technology, Aarhus, Denmark) and equilibrated at 37°C for 30 min in 5 mL of gassed (95% O_2_/5% CO_2_) PSS, pH 7.2. Vessels were then normalized to 90% of the internal circumference predicted to occur under a transmural pressure of 100 mmHg (Mulvany & Halpern, 1977). After normalization, vessels were left for 10 min and were then challenged with 60 mM KCl for 5 min. Endothelium integrity was assessed by stably precontracting vessels with 300 nM U46619, a thromboxane A2 mimetic (concentration producing approximately 90% of maximal contraction), followed by the addition of 10 μM carbachol (CCh). Vessels in which CCh‐induced relaxations were >90% of precontracted tone were designated as having a functional endothelium. Endothelium was removed by rubbing the intima layer with a human hair and CCh‐induced relaxations of <10% of precontracted tone indicated successful removal. All vessel segments used contained a functional endothelium unless otherwise stated. Vessel segments were incubated for 30 min in fresh PSS solution containing 1 mM CaCl_2_ and then precontracted with 300 nM U46619 or 60 mM KCl as required. This was followed by cumulative additions of CaCl_2_, increasing [Ca^2+^]_o_ from 1 to 10 mM. All inhibitors were added to the vessel segments 30 min before the construction of concentration–response curves to [Ca^2+^]_o_. All relaxant responses are expressed as percentage relaxation of tension induced by either 300 nM U46619 or 60 mM KCl. Data points on all graphs are mean values and error bars represent standard deviation. For each experiment *n* = number of animals, with multiple vessel segments used from each animal. Individual cumulative concentration–effect curves of [Ca^2+^]_o_ on U46619‐induced contractility were analyzed using a sigmoidal four‐parameter logistic equation using Graphpad Prism 6 software (GraphPad Software, Inc, San Diego, CA, USA) to extrapolate the effective concentration that produces 50% relaxation (EC_50_) or maximal effect at 10 mM [Ca^2+^]_o_ (*E*_max_). Mean EC_50_ and *E*_max_ values were compared using unpaired Students *t* test. Mean cumulative responses to increasing [Ca^2+^]_o_ were analyzed by two‐way ANOVA followed by Bonferroni post‐hoc tests. *p* < 0.05 was taken as statistically significant. Bonferroni comparisons are shown above the graph data points whereby *p < 0.05, **p < 0.01, ***p < 0.001, ****p < 0.0001 versus controls. Statistical analysis and graphs were made using Graphpad Prism 6 and Origin, version 6.1 (MicroCal Software, Northampton, MA, USA).

### 2.5 Materials

Mouse anti‐CaSR antibody (Abcam, ab19347), goat anti‐CD31 antibody (Santa Cruz, sc‐1506), rabbit anti‐CD31 (Abcam, ab182981), rabbit anti‐α‐actin antibody (Abcam, ab5694), rabbit anti‐synaptophysin antibody (Abcam, ab32127), and rabbit anti‐GPRC6A antibody (Novus, NL52576). The monoclonal mouse anti‐CaSR antibody used in the present work has been previously validated for the CaSR using siRNA (Mary et al., 2015), shRNA (Wei et al., 2015), and activating and knockdown CRISR technologies (Wu et al., 2019). All drugs were purchased from Sigma–Aldrich (Sigma Chemical Co., Poole, UK) or Tocris (Tocris Biosciences, Bristol, UK). Drugs were dissolved in distilled water (d.H_2_O), dimethyl sulfoxide (DMSO), or ethanol (EtOH).

## 3 RESULTS

### 3.1 The CaSR is expressed in rat mesenteric arteries

**FIGURE 1:**
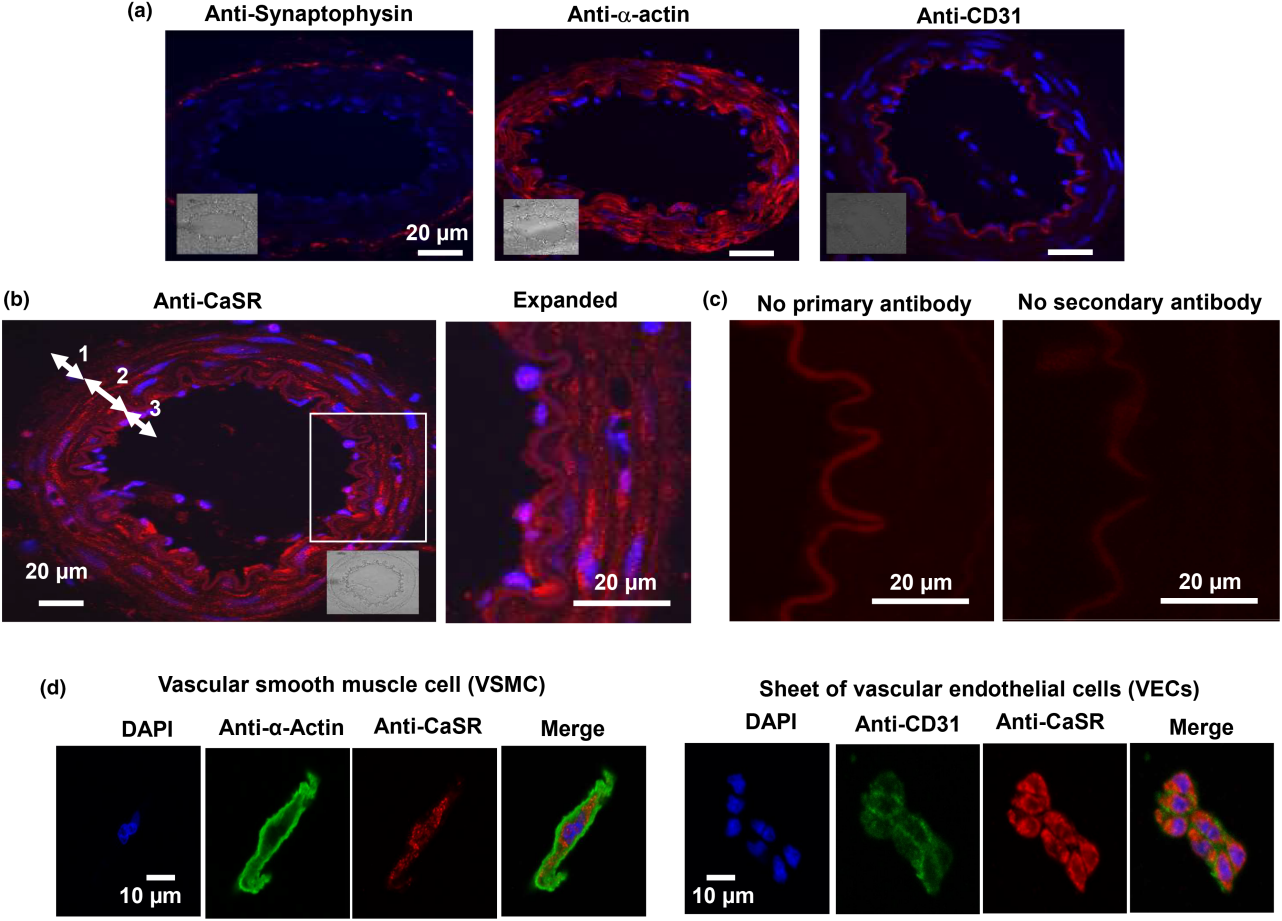
Expression of the calcium‐sensing receptor (CaSR) in rat mesenteric arteries. (a) Representative immunofluorescence images from three different first‐order rat mesenteric artery sections showing staining with anti‐synaptophysin, anti‐α‐actin, and anti‐CD31 antibodies as markers for synaptic terminals, vascular smooth muscle cells (VSMCs), and vascular endothelium cells (VECs). Blue staining presents 6‐diamidino‐2‐phenylindole (DAPI). (b) Immunofluorescent image showing staining of first‐order arterial section with anti‐CaSR antibody, with expanded image of arterial wall revealing staining at adventitia (1), medial (2), and intima (3) layers. (c) Control images showing lack of immunofluorescence, apart from autofluorescence at internal elastic lamina when either primary or secondary antibodies were omitted. (d) Immunofluorescence images showing expression of CaSRs in VSMCs and VECs, respectively.

In our initial experiments, we examined expression of the CaSR in arterial sections and freshly isolated VSMCs and VECs from first‐order rat mesenteric arteries using immunofluorescence techniques. Figure 1a shows that staining with antibodies for the synaptic terminal marker synaptophysin, the VSMCs marker α‐actin, and VECs marker CD31 revealed that arterial sections expressed synaptic terminals, VSMCs and VECs in the adventitia, media, and intima layers, respectively. In addition, Figure 1b demonstrates that staining with an anti‐CaSR antibody indicate that this receptor is present in each of these layers, and Figure 1c shows that no staining was present when either primary or secondary antibodies were omitted and that autofluorescence of the internal elastic lamina was observed at 555 nm wavelength as previously reported (Weston et al., 2008). Figure 1d also shows that CaSR staining was expressed in freshly isolated VSMCs and sheets of VECs, although there was limited staining at the plasma membrane in both cell types.

These findings clearly show that the CaSR is expressed in perivascular nerves, VSMCs, and VECs in rat mesenteric arteries.

### 3.2 Stimulation of the CaSR with increasing [Ca^2+^]_o_ induces vasorelaxations via endothelium‐dependent and independent pathways

**FIGURE 2:**
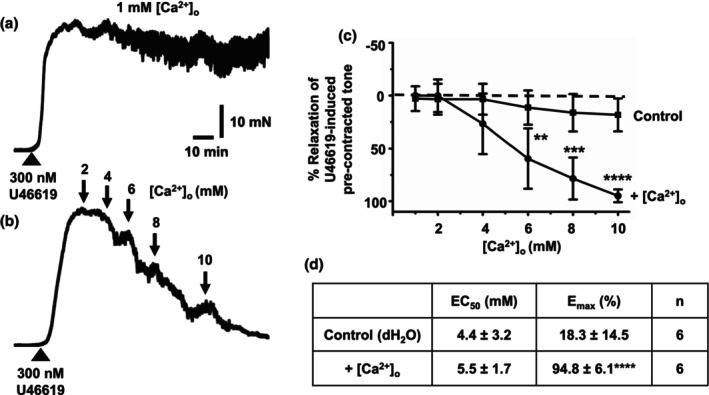
Effect of [Ca^2+^]_o_ on precontracted tone of rat mesenteric arteries. (a and b) Representative original traces showing the effect of [Ca^2+^]_o_ on precontracted tone by 300 nM U46619 in first‐order vessel segments. (a) U46619 produces a stable vasoconstriction in the presence of 1 mM [Ca^2+^]_o_. (b) The effect of increasing [Ca^2+^]_o_ from 1 to 10 mM. Both records are from vessel segments containing a functional endothelium. (c and d) Mean concentration‐effect curves and EC_50_ and E_max_ values showing the effect of increasing [Ca^2+^]_o_ on U46619‐induced precontracted tone in first‐order vessel segments. Mean values are ± standard deviation. Each data point is from *n* = number of animals, with multiple vessel segments per animal. Statistical analysis was carried out between control and effect of [Ca^2+^]_o_ on vessel segments. **p < 0.01, ***p < 0.001, ****p < 0.0001.

We investigated the functional role of the CaSR in rat mesenteric arteries by studying the effect of increasing [Ca^2+^]_o_ on precontracted tone evoked by 300 nM U46619 in first‐order vessels. In control experiments, Figure 2a shows that in 1 mM [Ca^2+^]_o_, 300 nM U46619 produced sustained tone. Figure 2b–d shows that increasing [Ca^2+^]_o_ from 1 to 10 mM induced concentration‐dependent relaxations of vessel segments compared with time‐matched vehicle controls, with almost complete relaxations observed with 10 mM [Ca^2+^]_o_, and a mean EC_50_ value of about 5–6 mM [Ca^2+^]_o_.

**FIGURE 3:**
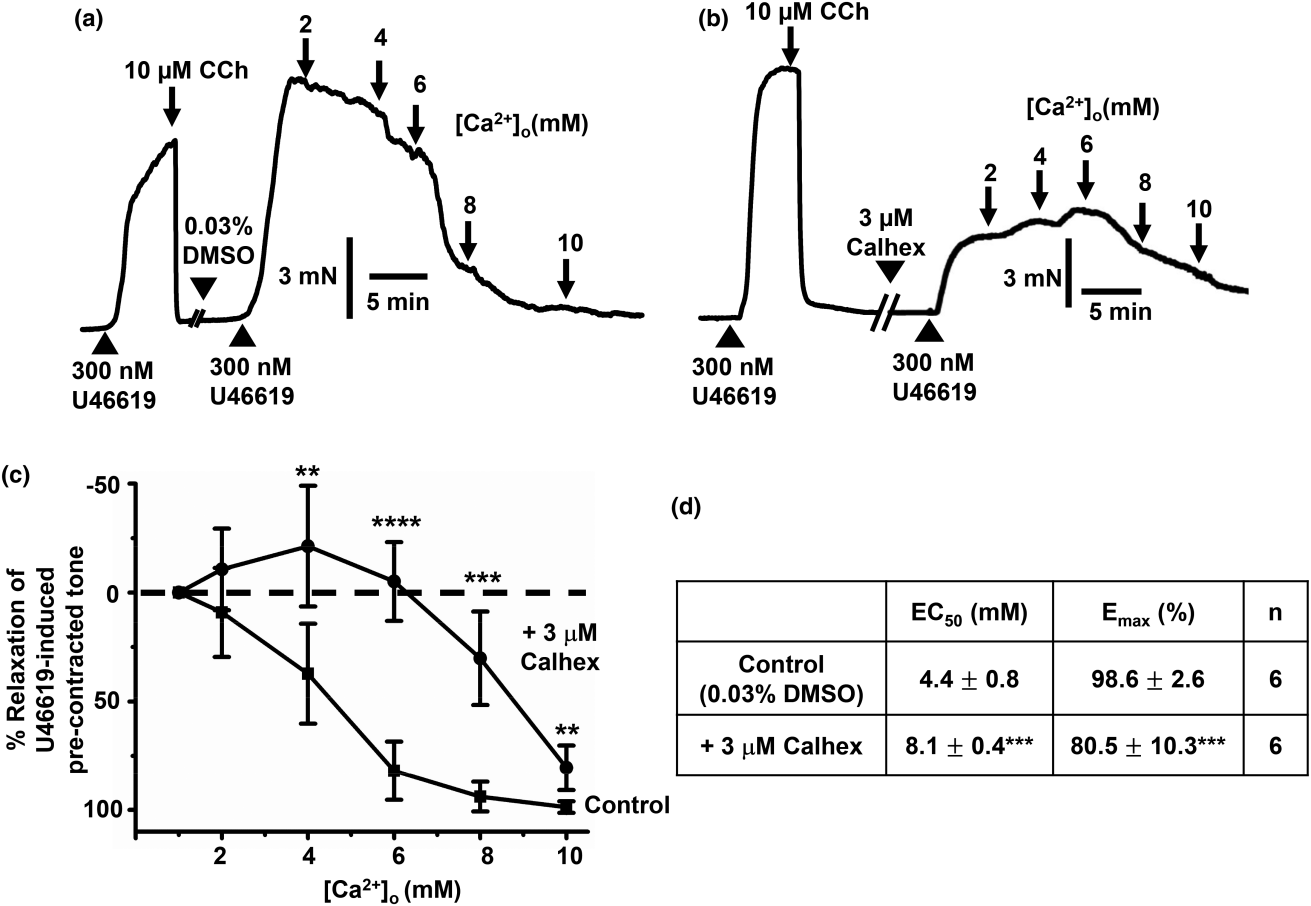
Effect of Calhex‐231 on [Ca^2+^]_o_‐induced vasorelaxations. (a and b) Representative original traces showing the effect of [Ca^2+^]_o_ on precontracted tone by 300 nM U46619 in the absence and presence of 3 μM Calhex‐231 in first‐order vessel segments, in which 10 μM CCh produced almost complete relaxations indicating both vessel segments contained a functional endothelium. (a) Following pretreatment with the control vehicle 0.03% DMSO for 20 min, U46619‐induced precontracted tone was inhibited by increasing [Ca^2+^]_o_ from 1 to 10 mM. (b) Following pretreatment with 3 μM Calhex‐231 for 20 min, increasing [Ca^2+^]_o_ from 1 to 10 mM was less effective at inhibiting U46619‐induced precontracted tone. It should be noted that pretreatment with 3 μM Calhex‐231 reduced peak precontracted tone produced by U46619. (c and d) Mean data showing that [Ca^2+^]_o_‐induced relaxations in first‐order vessel segments are reduced by pretreatment with 3 μM Calhex‐231. Mean values are ± standard deviation. Each data point is from *n* = number of animals, with multiple vessel segments per animal. Statistical analysis was carried out between effect of [Ca^2+^]_o_ on controls versus in the presence of Calhex‐231. **p < 0.01, ***p < 0.001, ****p < 0.0001.

To examine if [Ca^2+^]_o_‐induced vasorelaxations were mediated by the CaSR, we studied the effect of the negative allosteric modulator Calhex‐231 (Greenberg, Jahan, et al., 2016). Figure 3a–d shows that pretreatment with 3 μM Calhex‐231 produced a rightward shift in [Ca^2+^]_o_‐induced vasorelaxations of precontracted tone, producing an increase and decrease in mean EC_50_ and E_max_ values, respectively. In addition, Figure 3b shows that pretreatment with 3 μM Calhex‐231 reduced the peak amplitude of U46619‐induced precontracted, which likely represents the inhibitory effect of Calhex‐231 on voltage‐gated Ca^2+^ channels (VGCCs) as previously characterized (Greenberg, Jahan, et al., 2016).

**FIGURE 4:**
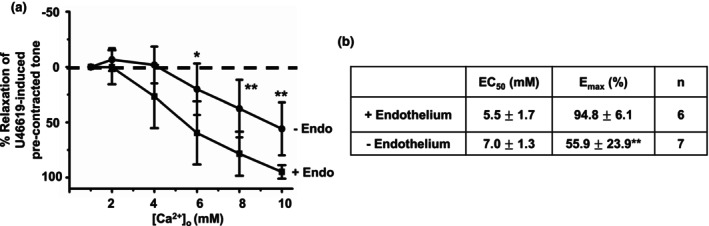
Role of endothelium in [Ca^2+^]_o_‐induced vasorelaxations. (a and b) Mean data showing that [Ca^2+^]_o_‐induced relaxations in first‐order vessel segments are reduced by removal of a functional endothelium. Mean values are ± standard deviation. Each data point is from *n* = number of animals, with multiple vessel segments per animal. Statistical analysis was carried out between effect of [Ca^2+^]_o_ on control versus removal of endothelium. *p < 0.05, **p < 0.01.

We studied if removal of a functional endothelium had an effect on [Ca^2+^]_o_‐induced vasorelaxations. Figure 4a,b reveals that removal of a functional endothelium, determined by the absence of CCh‐evoked relaxations of precontracted tone (see Section 2), partially inhibited [Ca^2+^]_o_‐mediated vasorelaxations with a rightward shift in mean EC_50_ values and reduction in E_max_ values.

These results provide evidence that increasing [Ca^2+^]_o_ induces relaxation of rat mesenteric arteries through stimulation of the CaSR and that this likely involves both endothelium‐dependent and ‐independent pathways.

### 3.3 NO—but not cyclooxygenase—or prostacyclin receptor‐mediated pathways contribute to [Ca^2+^]_o_‐mediated vasorelaxations

In the next series of experiments, we investigated the role of potential endothelium‐dependent relaxant mechanisms involved in mediating [Ca^2+^]_o_‐induced vasorelaxations, namely NO, cyclooxygenase, and prostacyclin receptors, which are all established molecules involved in endothelium‐dependent vasorelaxations.

**FIGURE 5:**
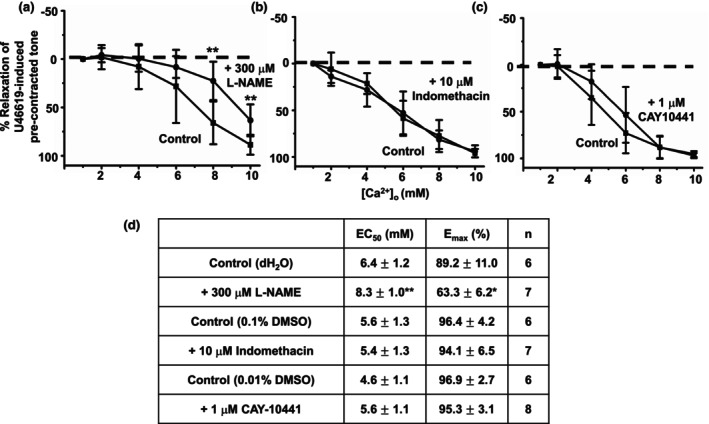
Role of NO, cyclooxygenase, and prostacyclin receptors in [Ca^2+^]_o_‐induced vasorelaxations. (a and d), Mean data showing that [Ca^2+^]_o_‐induced relaxations in first‐order vessel segments are reduced by pretreatment with the eNOS inhibitor L‐NAME (300 μM). (b, c and d) Mean data showing that [Ca^2+^]_o_‐induced relaxations in first‐order vessel segments were unaffected by pretreatment with the cyclooxygenase inhibitor indomethacin (10 μM) and the prostacyclin receptor inhibitor CAY10441 (1 μM). Mean values are ± standard deviation. Each data point is from *n* = number of animals, with multiple vessel segments per animal. Statistical analysis was carried out between effect of [Ca^2+^]_o_ on controls versus 300 μM L‐NAME. *p < 00.05, **p < 0.01.

Figure 5a,d demonstrates that pretreatment with the endothelium NO synthase (eNOS) inhibitor, 300 μM L‐NAME, inhibited [Ca^2+^]_o_‐induced vasorelaxations of precontracted tone. In contrast, Figure 5b–d shows that pretreatment with the cyclooxygenase (COX) inhibitor, 10 μM indomethacin, and the prostacyclin receptor blocker, 1 μM CAY10441 had no effect on [Ca^2+^]_o_‐induced vasorelaxations of precontracted tone.

These findings suggest that NO production, but not COX‐mediated products or prostacyclin receptors, is likely to be involved in mediating [Ca^2+^]_o_‐induced vasorelaxations produced by endothelium‐dependent mechanisms.

### 3.4 Hyperpolarizations underlie [Ca^2+^]_o_‐mediated vasorelaxations

**FIGURE 6:**
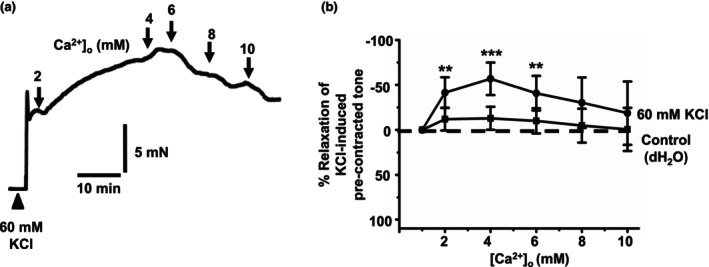
Effect of [Ca^2+^]_o_ on precontracted tone by KCl in rat mesenteric arteries. (a and b) Representative original trace and mean data showing that increasing [Ca^2+^]_o_ from 1 to 10 mM did not induce vasorelaxations of precontracted tone by 60 mM KCl in first‐order vessel segments. Mean values are ± standard deviation. Each data point is from *n* = 7 animals, with multiple vessel segments per animal. Statistical analysis was carried out between time‐matched control versus effect of [Ca^2+^]_o_ on KCl‐induced contractions. **p < 0.01, ***p < 0.001.

It has been proposed that hyperpolarizations of VSMCs by BK_Ca_ channels‐ and IK_Ca_ channels‐mediated processes underlie CaSR‐induced vasorelaxations in rat and rabbit mesenteric arteries (Awumey et al., 2008; Greenberg, Jahan, et al., 2016; Greenberg, Shi, et al., 2016; Ishioka & Bukoski, 1999; Weston et al., 2005). We tested this proposal by studying the effect of [Ca^2+^]_o_ on precontracted tone induced by 60 mM KCl in vessel segments of first‐order rat mesenteric arteries containing a functional endothelium. Application of KCl clamps the membrane potential at about −20 mV and therefore induces contraction solely by Ca^2+^ influx through activation of VGCCs. Figure 6a,b reveals that increasing [Ca^2+^]_o_ from 1 to 10 mM did not induce vasorelaxations of precontracted tone by KCl, but instead potentiated precontracted tone compared with time‐matched controls. These findings provide evidence that hyperpolarization mechanisms are likely pivotal in mediated [Ca^2+^]_o_‐induced vasorelaxations.

### 3.5 BK_Ca_ channels‐ and K_ATP_ channels‐mediated [Ca^2+^]_o_ induce vasorelaxations via endothelium‐dependent and ‐independent pathways, respectively

In our final series of experiments, we investigated the effect of inhibitors of K^+^ channel subtypes, known to contribute to hyperpolarization and relaxation of the vasculature, on [Ca^2+^]_o_‐induced vasorelaxations of precontracted tone.

**FIGURE 7:**
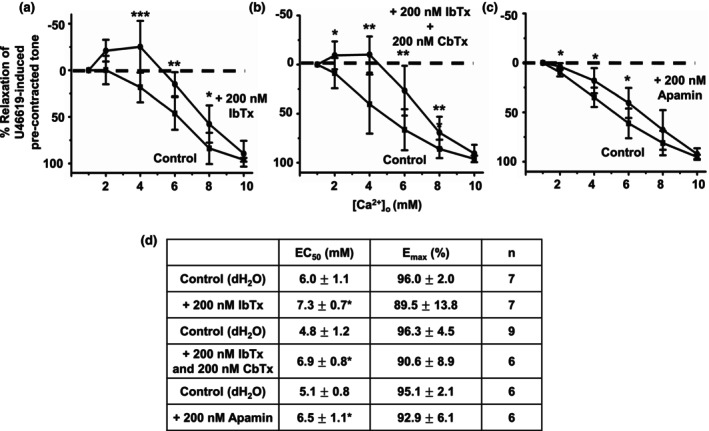
Effect of Ca^2+^‐activated K^+^ channel inhibitors on [Ca^2+^]_o_‐induced vasorelaxations. (a, b, and d) Mean data showing that pretreatment with 200 nM IbTx or 200 nM IbTx and 200 nM CbTx had a similar inhibitory effect on [Ca^2+^]_o_‐induced vasorelaxations in first‐order vessels. (c and d) Pretreatment with 200 nM apamin also had a small but significant inhibitory effect on [Ca^2+^]_o_‐induced vasorelaxations. Mean values are ± standard deviation. Each data point is from *n* = number of animals, with multiple vessel segments per animal. Statistical analysis was carried out between effect of [Ca^2+^]_o_ controls versus pretreatment with inhibitor(s). *p < 0.05, **p < 0.01, ***p < 0.001.

First, we studied the action of iberotoxin (IbTx), charybdotoxin (CbTx), and apamin that are well‐characterized inhibitors of BK_Ca_ channels, BK_Ca_ channels/IK_Ca_ channels, and SK_Ca_ channels, respectively, on [Ca^2+^]_o_‐induced vasorelaxations. Figure 7a,b,d shows that pretreatment of vessel segments with 200 nM IbTx reduced [Ca^2+^]_o_‐induced vasorelaxations and that pretreatment with a combination of both 200 nM IbTx and 200 nM CbTx produced no further inhibition than IbTx alone. Figure 7c,d show that pretreatment of 200 nM apamin also had a small but significant inhibitory action on [Ca^2+^]_o_‐induced vasorelaxations.

**FIGURE 8:**
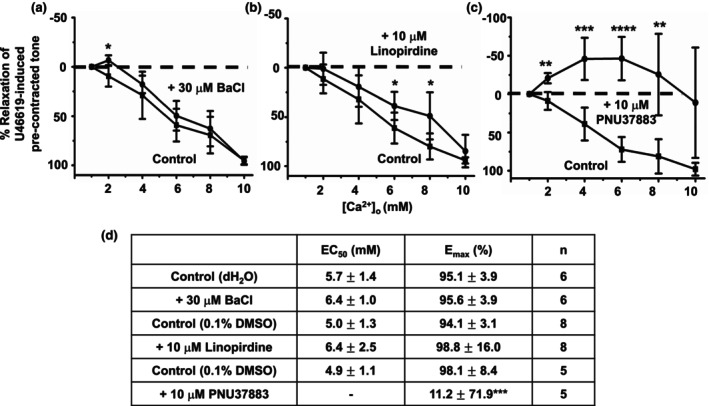
Effect of K^+^ channel inhibitors on [Ca^2+^]_o_‐induced vasorelaxations. (a, b, and d) Mean data showing that pretreatment with the K_ir_ channel inhibitor BaCl_2_ (30 μM) and the Kv7 channel inhibitor linopridine (10 μM) had small but significant inhibitory actions on [Ca^2+^]_o_‐induced vasorelaxations in first‐order vessels, whereas (c and d) show that they were completely abolished by pretreatment with the K_ATP_ channel inhibitor PNU37883 (10 μM). Moreover, in the presence of PNU37883, increasing [Ca^2+^]_o_ produce a significant increase in precontracted tone. Mean values are ± standard deviation. Each data point is from *n* = number of animals, with multiple vessel segments per animal. Statistical analysis was carried out between effect of [Ca^2+^]_o_ controls versus pretreatment with inhibitor(s). *p < 0.05, **p < 0.01, ***p < 0.001, ****p < 0.0001.

Figure 8a,b,d shows that pretreatment with the K_ir_ channel and Kv7 channel inhibitors, 30 μM BaCl_2_, and 10 μM linopirdine, respectively, had small but significant inhibitory effects on [Ca^2+^]_o_‐induced vasorelaxations. In contrast, Figure 8c,d reveals that pretreatment with the K_ATP_ channel inhibitor, 10 μM PNU37883, completely abolished [Ca^2+^]_o_‐induced vasorelaxations such that increasing [Ca^2+^]_o_ between 1 mM and 8 mM now augmented precontracted tone. Attempts to use glibenclamide, another K_ATP_ blocker, to investigate the role of K_ATP_ channels in [Ca^2+^]_o_‐induced vasorelaxations failed (data not shown) due to this blocker inhibiting U46619‐induced precontracted tone as previously described (Cocks et al., 1990).

**FIGURE 9:**
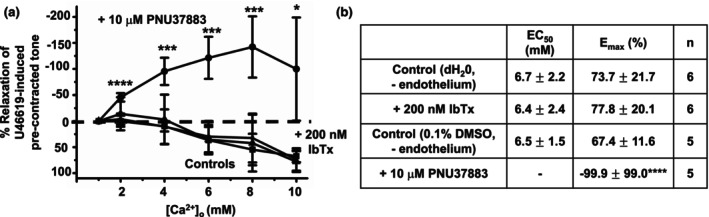
Effect of IbTx and PNU37883 on [Ca^2+^]_o_‐induced vasorelaxations in endothelium‐removed rat mesenteric arteries. (a and b) Mean data showing that pretreatment with the 200 nM IbTx had no effect, whereas 10 μM PNU37883 completely abolished [Ca^2+^]_o_‐induced relaxations in first‐order vessel segments without a functional endothelium. In the presence of PNU37883, increasing [Ca^2+^]_o_ produce a significant increase in precontracted tone. Mean values are ± standard deviation. Each data point is from *n* = number of animals, with multiple vessel segments per animal. Statistical analysis was carried out between effect of [Ca^2+^]_o_ controls versus pretreatment with inhibitor(s). *p < 0.05, ***p < 0.001, ****p < 0.0001.

These findings indicate that BK_Ca_ channels and K_ATP_ channels have important roles in mediated [Ca^2+^]_o_‐induced vasorelaxations in rat mesenteric arteries. Therefore, we further studied whether BK_Ca_ channels and K_ATP_ channels are involved in endothelium‐dependent or ‐independent pathways of [Ca^2+^]_o_‐induced vasorelaxations. Figure 9a,b clearly shows that in the absence of a functional endothelium, [Ca^2+^]_o_‐induced vasorelaxations were unaffected by pretreatment with 200 nM IbTx, but that pretreatment with 10 μM PNU37883 completed abolished [Ca^2+^]_o_‐induced vasorelaxations and that increasing [Ca^2+^]_o_ now produced a substantial increase in precontracted tone. These findings suggest that BK_Ca_ channels and K_ATP_ channels are involved in [Ca^2+^]_o_‐induced vasorelaxations through distinct endothelium‐dependent and ‐independent pathways, respectively.

## 4 DISCUSSION

This study shows that stimulation of the CaSR by increasing [Ca^2+^]_o_ induces relaxations in rat mesenteric arteries that involve multiple pathways including an endothelium‐dependent pathway involving NO production that stimulates BK_Ca_ channels in VSMCs and an endothelium‐independent pathway involving stimulation of K_ATP_ channels in VSMCs.

### 4.1 CaSRs are expressed in perivascular nerves, VSMCs, and VECs

Our immunofluorescence studies show that sections from first‐order rat mesenteric arteries contain adventitia, media, and intima layers composed of perivascular nerves, VSMCs, and VECs, respectively, and that the CaSR is expressed in each of these layers and cell types. In addition, we also show that the CaSR is present in freshly isolated single VSMCs and sheets of VECs.

Our findings confirm earlier immunofluorescence studies which show expression of the CaSR in perivascular nerves, VSMCs, and VECs in rat and rabbit mesenteric arteries (Bukoski et al., 1997; Greenberg, Shi, et al., 2016; Mupanomunda et al., 1998; Wang & Bukoski, 1998; Weston et al., 2005), and in VSMCs and VECs in mouse aorta (Loot et al., 2013) and human arteries (Molostvov et al., 2007). It is interesting to note that the cellular distribution of CaSR staining with an anti‐CaSR antibody in our studies exhibited relatively stronger cytosolic staining signals compared to the plasma membrane in VSMCs and VECs, which is different from our previous study using a similar approach in rabbit mesenteric VSMCs and VECs (Greenberg, Shi, et al., 2016).

### 4.2 CaSR‐mediated vasorelaxations induced by [Ca^2+^]_o_ are partially endothelium‐dependent involving NO production

Increasing [Ca^2+^]_o_ from 1 to 10 mM induced a concentration‐dependent relaxations of precontracted tone by a thromboxane agonist in first‐order rat mesenteric arteries, which had an EC_50_ value of about 5–6 mM [Ca^2+^]_o_ and almost full relaxation at 10 mM [Ca^2+^]_o_. In the presence of the allosteric calcilytic Calhex‐231, [Ca^2+^]_o_‐induced vasorelaxations were less sensitive to [Ca^2+^]_o_, with concentration–response curves shifted to the right and increasing mean EC_50_ and decreasing mean E_max_ values. These findings confirm earlier studies which showed [Ca^2+^]_o_‐induced relaxations in rat, rabbit, and mouse mesenteric arteries (Awumey et al., 2008, 2013; Bukoski et al., 1997, 2002; Dora et al., 2008; Greenberg et al., 2017, 2019; Greenberg, Jahan, et al., 2016; Greenberg, Shi, et al., 2016; Ishioka & Bukoski, 1999; Mupanomunda et al., 1998, 1999; Ohanian et al., 2005; Thakore & Ho, 2011; Wang & Bukoski, 1998; Weston et al., 2005, 2008) Taken together, these findings indicate that [Ca^2+^]_o_‐induced vasorelaxations are likely to be mediated by the CaSR. However, we recognize that Calhex‐231 did not abolish vasorelaxations to [Ca^2+^]_o_, particularly at higher [Ca^2+^]_o_, and therefore it is possible other [Ca^2+^]_o_‐induced mechanisms may contribute to these responses. For example, [Ca^2+^]_o_ is proposed to modulate K_ir_ channel and Na^+^/K^+^‐ATPase activities that are known to modulate vasorelaxations (Hangaard et al., 2015). In addition, Calhex‐231 reduced precontracted tone induced by U46619 which is likely due to an inhibitory action on VGCCs as previously characterized (Greenberg, Shi, et al., 2016) that may also influence [Ca^2+^]_o_‐induced responses.

In contrast to our findings, previous studies have indicated that [Ca^2+^]_o_ relaxed first‐ and second‐order rat mesenteric arteries precontracted with α_1_‐adrenoceptors stimulants noradrenaline or phenylephrine with an EC_50_ of about 2–3 mM with 5–6 mM producing near full relaxation (Bukoski et al., 1997; Ishioka & Bukoski, 1999; Mupanomunda et al., 1998, 1999; Wang & Bukoski, 1998). It is unclear why in the present study, vasorelaxations are less sensitive to [Ca^2+^]_o_ than in previous studies carried out in the same vascular bed. This may be due to the present study using the thromboxane receptor agonist U46619 to produce precontracted tone instead of α_1_‐adrenoceptors stimulants.

An important question is whether [Ca^2+^]_o_‐induced vasorelaxations with an EC_50_ of 5–6 mM are physiological when plasma [Ca^2+^]_o_ is closely regulated between 1 and 2 mM (Brown & MacLeod, 2001). It is proposed that the CaSR in rat mesenteric arteries are likely to be activated by paracrine Ca^2+^ signaling within interstitial spaces produced by active Ca^2+^ extrusion systems in VSMCs and VECs such as the plasma membrane Ca^2+^‐ATPase and Na^+^/Ca^2+^ exchanger (Dora et al., 2008). For comparison, rises of K^+^ to over 10 mM have been recorded in interstitial spaces from rat hepatic and mesenteric arteries due to the opening of K^+^ channels and changes in Na^+^/K^+^‐ATPase activity which leads to regulation of vascular tone (Edwards et al., 1998; Weston et al., 2008). It is therefore possible that localized [Ca^2+^]_o_ may indeed rise to 5–10 mM to produce a significant impact on vasorelaxations as described in the current work. In the future, it would be useful to measure [Ca^2+^]_o_ changes in the interstitial spaces of the vasculature using Ca^2+^‐selective microelectrodes (Messerli & Smith, 2010).

Our data also show that [Ca^2+^]_o_‐induced vasorelaxations were partially reduced by the removal of a functional endothelium and the eNOS inhibitor L‐NAME. These results support earlier studies showing a contributory role for the endothelium in mediating [Ca^2+^]_o_‐induced relaxations in rat mesenteric arteries (Awumey et al., 2008; Dora et al., 2008) but are in contrast to studies in rabbit and mouse mesenteric arteries (Greenberg et al., 2017, 2019; Greenberg, Shi, et al., 2016) and mouse aorta (Loot et al., 2013) in which removal of endothelium completely abolished [Ca^2+^]_o_‐induced vasorelaxations. Moreover, L‐NAME did not inhibit [Ca^2+^]_o_‐induced vasorelaxations in the rat mesenteric arteries (Bukoski et al., 1997) but markedly inhibited both [Ca^2+^]_o_‐induced vasorelaxations and ‐NO production and in VECs in rabbit and mouse mesenteric arteries (Greenberg et al., 2017, 2019; Greenberg, Shi, et al., 2016) and mouse aorta (Loot et al., 2013). In addition, L‐NAME inhibited NO production induced by the CaSR stimulant spermine in human aortic VECs (Ziegelstein et al., 2006).

Taken together, our findings indicate that activation of the CaSR, by increasing [Ca^2+^]_o_, induces relaxation of rat mesenteric arteries through both an endothelium‐dependent pathway involving NO production and an endothelium‐independent pathway. The present work does not identify, where the CaSR stimulated by [Ca^2+^]_o_ is located, but it is likely to be either at perivascular nerves or VECs. Previous evidence has suggested that stimulation of the CaSR in perivascular nerves (Awumey et al., 2008; Bukoski et al., 1997, 2002; Ishioka & Bukoski, 1999; Mupanomunda et al., 1998, 1999; Wang & Bukoski, 1998) are important in rat mesenteric arteries, although this contrasts to other findings which show that CaSR‐mediated hyperpolarizations leading to relaxations in rat mesenteric arteries require VECs (Weston et al., 2005, 2008). In addition, the CaSR in VECs is essential in rabbit and mouse mesenteric arteries (Greenberg et al., 2017, 2019; Greenberg, Shi, et al., 2016). The question of where [Ca^2+^]_o_‐activated receptors are located will need to be further addressed in future work, but with a significant contribution of the [Ca^2+^]_o_‐induced vasorelaxations being endothelium‐independent it does raise the likelihood of perivascular nerve involvement and release of vasoactive substances, such as anandamide, calcitonin‐gene‐related peptide, substance P, as previously indicated by the work of Bukoski's group (see earlier citations). Moreover, the potential for species differences in functional expression of the CaSR in the vasculature affirms the importance of investigating these actions in the human vasculature (Molostvov et al., 2007).

### 4.3 [Ca^2+^]_o_‐induced vasorelaxations involve hyperpolarization and BK_Ca_ channels and K_ATP_ channels

Previous works have indicated that activation of IK_Ca_ channels on VECs and BK_Ca_ channels on VSMCs are important in mediating CaSR‐induced hyperpolarizations of VSMCs and vasorelaxations (Awumey et al., 2008; Greenberg, Shi, et al., 2016; Weston et al., 2008). The present further confirms the importance of CaSR‐induced hyperpolarizations in mediating vasorelaxations by showing that [Ca^2+^]_o_ does not induce relaxation of KCl‐induced precontracted tone in which membrane potential is likely to be clamped at about −20 mV as previously described (Bukoski et al., 2002; Greenberg, Shi, et al., 2016).

To identify possible K^+^ channels involved in mediating [Ca^2+^]_o_‐induced vasorelaxations, we produced a pharmacological profile using established inhibitors of different K^+^ channel subtypes. Our findings conclude that BK_Ca_ channels and K_ATP_ channels are likely to have predominant roles in mediating [Ca^2+^]_o_‐induced vasorelaxations, with smaller contributory roles for SK_Ca_ channels, K_ir_ channels, and Kv7 channels. In contrast to earlier work, we did not find a role for IK_Ca_ channels (Greenberg, Shi, et al., 2016; Weston et al., 2005) as the magnitude of inhibition of [Ca^2+^]_o_‐induced vasorelaxations by the BK_Ca_ channel inhibitor IbTx alone was similar to that produced by combining IbTx and the BK_Ca_ channel/IK_Ca_ channel blocker CbTx.

The effect of inhibiting K_ATP_ channels with PNU37883 was particularly pronounced, revealing marked [Ca^2+^]_o_‐induced vasoconstrictions. This augmentation of precontracted tone by [Ca^2+^]_o_ is likely due to stimulation of the CaSR on VSMCs leading to activation of Gq‐mediated pathways as previously proposed (Greenberg, Shi, et al., 2016; Li et al., 2011; Ohanian et al., 2005; Schepelmann et al., 2016; Wonneberger et al., 2000).

BK_Ca_ channels are generally thought to be expressed in the vasculature on VSMCs although there is evidence that they may also be present on VECs (Sandow & Grayson, 2009), whereas K_ATP_ channels are known to reside on both VSMCs and VECs. Therefore, we investigated the role of these K^+^ channels in [Ca^2+^]_o_‐induced endothelium‐dependent and ‐independent vasorelaxations by testing the effect of inhibitors on responses in the absence of a functional endothelium. As predicted, inhibition of BK_Ca_ channels had no further inhibitory action on [Ca^2+^]_o_‐induced vasorelaxations after removal of an endothelium, which likely indicates that stimulation of eNOS, NO production, and activation of BK_Ca_ channels in VSMCs play a significant role in the endothelium‐dependent pathway that supports earlier studies in rabbit and mouse mesenteric arteries (Greenberg et al., 2017, 2019; Greenberg, Shi, et al., 2016). However, since BKCa are reported to be expressed in VECs (Sandow & Grayson, 2009), we cannot exclude the possibility that BK_Ca_ channels may contribute to endothelium‐dependent [Ca^2+^]_o_‐mediated vasorelaxations. Interestingly, inhibition of K_ATP_ channels still produced a profound inhibition of [Ca^2+^]_o_‐induced vasorelaxations and revealed [Ca^2+^]_o_‐induced vasoconstrictions, in the absence of a functional endothelium. These findings indicate that K_ATP_ channels on VSMCs, perhaps stimulated by perivascular nerve‐mediated CaSR responses, contribute to endothelium‐independent responses to [Ca^2+^]_o_. Interestingly, the K_ATP_ channel inhibitor glibenclamide was previously found to have no effect on [Ca^2+^]_o_‐induced relaxations in rat mesenteric arteries precontracted with α_1_‐adrenoceptor agonists (Ishioka & Bukoski, 1999). As such, the current work provides the first evidence that K_ATP_ channels have been proposed to be involved in CaSR‐mediated responses in the vasculature, and at this stage, it is unclear why our results differ from previous work.

## 5 CONCLUSION

The present data clearly indicate that stimulation of the CaSR by increasing [Ca^2+^]_o_ induces relaxations of isolated segments of first‐order rat mesenteric arteries using wire myography. Our findings suggest that [Ca^2+^]_o_‐induced vasorelaxations occur via multiple pathways including an endothelium‐dependent pathway involving NO production and activation of BK_Ca_ channels in VSMCs and an endothelium‐independent pathway involving K_ATP_ channels on VSMCs. The location of the CaSR involved in these vasorelaxations is unclear, but it is likely to be on perivascular nerves and/or VECs. In addition, further work is needed to understand the difference between current and previous work on cellular pathways involved in mediating [Ca^2+^]_o_‐induced vasorelaxations. Characterization of the action of the CaSR in the human vasculature will be important.

## AUTHOR CONTRIBUTIONS

S.R.E.C.C, H.Z.E.G, E.J.C, and P.Z performed and analyzed experiments. S.R.E.C.C, H.Z.E.G, I.A.G, and A.P.A conceived the experimental design. A.P.A. wrote the manuscript. All authors contributed to the preparation of the manuscript and critically advised and agreed to the final submitted article.

## FUNDING INFORMATION

This work was supported by a British Heart Foundation PhD Studentships for H. Z. E. Greenberg (FS/13/10/30021 to A.P.A) and S.R.E.C‐C (FS/17/40/32942 to A.P.A) and by the Biotechnology and Biological Sciences Research Council (BB/J007226/1 to A.P.A).

## CONFLICT OF INTEREST STATEMENT

None Declared.

## Data Availability

The data that support the findings of this study are available from the corresponding author upon reasonable request.
